# A Meta-Analysis and Systematic Review of Normothermic and Hypothermic Machine Perfusion in Liver Transplantation

**DOI:** 10.3390/jcm12010235

**Published:** 2022-12-28

**Authors:** Joseph Mugaanyi, Lei Dai, Changjiang Lu, Shuqi Mao, Jing Huang, Caide Lu

**Affiliations:** 1 Department of Hepato-Pancreato-Biliary Surgery, Ningbo Medical Center Li Huili Hospital, The Affiliated Hospital of Ningbo University, Ningbo 315040, China; 2 School of Medicine, Ningbo University, Ningbo 315211, China

**Keywords:** machine perfusion, normothermic, hypothermic, liver transplant, survival

## Abstract

Background: The gap between the demand and supply of donor livers is still a considerable challenge. Since static cold storage is not sufficient in marginal livers, machine perfusion is being explored as an alternative. The objective of this study was to assess (dual) hypothermic oxygenated machine perfusion (HOPE/D-HOPE) and normothermic machine perfusion (NMP) in contrast to static cold storage (SCS). Methods: Three databases were searched to identify studies about machine perfusion. Graft and patient survival and postoperative complications were evaluated using the random effects model. Results: the incidence of biliary complications was lower in HOPE vs. SCS (OR: 0.59, 95% CI: 0.36–0.98, *p* = 0.04, *I*^2^: 0%). There was no significant difference in biliary complications between NMP and SCS (OR: 0.76, 95% CI: 0.41–1.40, *p* = 0.38, *I*^2^: 55%). Graft and patient survival were significantly better in HOPE than in SCS (HR: 0.40, 95% CI: 0.23–0.71, *p* = 0.002, *I*^2^: 0%) and (pooled HR: 0.43, 95% CI: 0.20–0.93, *p* = 0.03, *I*^2^: 0%). Graft and patient survival were not significantly different between NMP and SCS. Conclusion: HOPE/D-HOPE and NMP are promising alternatives to SCS for donor liver preservation. They may help address the widening gap between the demand for and availability of donor livers by enabling the rescue and transplantation of marginal livers.

## 1. Introduction

Although the number of liver transplants performed globally has increased yearly, the availability of donor organs is overshadowed by the demand. More and more centers have optimized and adopted the use of extended criteria donor (ECD) organs to narrow the gap [1,2]. However, ECD organs are more susceptible to ischemia-reperfusion injury and have an increased mortality risk than standard criteria donor organs [3]. Static cold storage (SCS) is the gold-standard method for preserving donor livers. Although SCS has good outcomes for optimal livers, especially donation after brain death (DBD), it has been reported as insufficient in suboptimal livers, with a high risk for complications [4,5,6]. To address the limitations of SCS, centers worldwide have investigated the use of dynamic preservation of livers using machine perfusion ex situ. Two types of machine perfusion are utilized in the clinical preservation of donor livers: normothermic machine perfusion (NMP) and (dual) hypothermic oxygenated machine perfusion (HOPE/D-HOPE) [7,8,9]. Normothermic machine perfusion is initiated immediately after standard organ procurement to replace cold storage [10,11,12,13,14,15]. Unlike NMP, which keeps the liver continuously perfused close to or at normal core temperature, HOPE/D-HOPE involves continuous perfusion of the liver with a cooled, oxygenized perfusate [11,16,17,18,19]. HOPE has been associated with improved graft function compared to SCS [18,20,21,22].

Although numerous studies have explored the dynamic preservation of livers over the past two decades using machine perfusion (NMP or HOPE/D-HOPE) compared to SCS in clinical settings, the majority are small sample-size studies. Based on current literature, it is not very clear which may be comparatively better between HOPE/D-HOPE and NMP when compared with SCS, which is the standard method for preserving donor livers. Ischemia re-perfusion injury is one of the main concerns in SCS. Ischemia re-perfusion injury affects graft survival, which influences patient survival. Machine perfusion aims to address this problem. The occurrence of postoperative complications also has an impact on patient survival. Therefore, in this systematic review and meta-analysis, our primary objective is to assess and compare patient and graft survival in liver transplant patients after ex situ machine perfusion compared to SCS. The secondary objective is to evaluate the occurrence of postoperative complications after liver transplantation.

## 2. Methods

### 2.1. Search Strategy

The PubMed, Web of Science and Scopus databases were queried for studies reporting on normothermic and hypothermic machine perfusion in liver transplantation through September 2022. The full search syntax for each database is documented in the Supplementary Materials. Full-text studies reporting on NMP or HOPE with an SCS control group were included. Abstracts, reviews, case reports, editorials and letters and non-English language studies were excluded. First, studies were evaluated for inclusion based on the title and abstract. Studies were subsequently included based on a review of the study’s full text. The selection was carried out by two independent reviewers (MJ and DL). The final article inclusion was based on a mutual consensus of the two reviewers. Cross-referencing was performed on the studies to identify any other related studies. Studies comparing either NMP or HOPE to SCS were included; studies that compared NRP, SCS and NMP/HOPE were also included. The most recent study was included if multiple studies reported results from the same source. This manuscript was prepared according to the Cochrane guidelines for interventional system reviews and the PRISMA statement (Preferred Reporting Items for Systematic Reviews and Meta-Analyses) [23,24].

### 2.2. Quality Assessment

Two independent reviewers performed the quality assessment of all the studies included in the meta-analysis. The evaluation was according to the Downs and Black checklist [25]. We used the modified Downs and Black checklist composed of 5 categories (quality of reporting, external validity, potential for bias, confounding and power analysis). For each study, the maximum possible score is 32 points. Most studies reporting on machine perfusion in liver transplantation have small sample sizes. To address this issue, the last item (study power) was modified from a 5-point scale to assign 5 points if there was adequate study power, 3 if the study power was calculated, and 1 if there was no study power calculation. 

### 2.3. Data Extraction

Data were extracted independently by the two reviewers using standardized forms. Baseline and outcome data were extracted for the research (NMP/HOPE) and control (CSC) groups. Baseline data includes sample size in each group, age, donor type and BMI. Outcome data includes graft survival, patient survival, biliary complications, hospital stay, vascular complications and primary non-function. Data were collected, aggregated and reported. For studies that did not report survival data, the data were extracted from Kaplan–Meier survival curves using methods described by Tierney et al. [26].

### 2.4. Statistical Analysis

Pooling of available outcome data (biliary complications, vascular complications, graft survival, patient survival, hospital stay and primary non-function) was performed using “Review Manager 5.3” using the random effects model. Study heterogeneity was quantified using the DerSimonian–Laird method. The pooled data were presented with their corresponding 95% confidence intervals (CI). The graft and patient survival between the groups were compared using generic inverse variance described by Tierney et al. [26]. The hazard ratios were reported with the respective 95% CI and corresponding forest plots used for visual reporting. The random effects model was used for biliary complications, vascular complications, hospital stay and primary non-function, and the odds ratios (OR) with 95% CI were reported on forest plots. Study heterogeneity was assessed using the I^2^ statistic. *p*-value < 0.05 was considered significant.

## 3. Results

### 3.1. Literature Search Results

**Figure 1:**
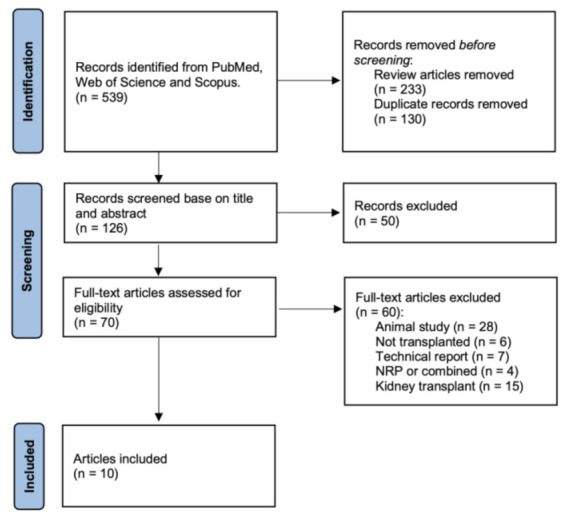
PRISMA flow chart.

A text search was performed on 13 September 2022. A PRISMA flow chart of the search process is presented in Figure 1. Upon initial search, 529 results were returned, and 70 articles were selected for full-text assessment. Finally, 10 articles were included in the analysis [7,11,18,20,27,28,29,30,31,32]. The quality assessment of all the included studies is summarized in Table 1. The studies were of moderately good quality; the median score was 20 out of 32 points (range 17–23). Three studies had DCD and DBD donors in the analyzed [7,11,27]. Dutkowski et al. compared DCD HOPE to DCS SCS and DBD SCS [29]. Gaurav et al. compared SCS, NMP and NRP [30]; only SCS and NMP data were included. Vascular complications were reported in eight studies [11,18,20,28,29,30,31,32], PNF in six [7,11,18,29,30,31], biliary complications in nine [11,18,20,27,28,29,30,31,32] and hospital stay in eight [7,11,20,27,28,29,30,31]. Seven studies reported adequate data to compare patient survival [11,18,20,28,30,31,32], and nine to compare graft survival [7,11,18,20,28,29,30,31,32]. The baseline demographic and clinical data of the included studies are summarized in Table 2. In total, 1104 liver transplant recipients were included (504 machine-perfused livers and 600 static cold-storage livers) in this study. Of the 504 perfused livers, 371 were NMP and 133 were HOPE. In one study, HOPE was combined with NRP [28]. Three studies only reported patient survival rates without sufficient data to extract survival data [7,27,29]. Bral et al. did not provide sufficient data to extract graft survival data [27].

### 3.2. Complications after Liver Transplant

**Figure 2:**
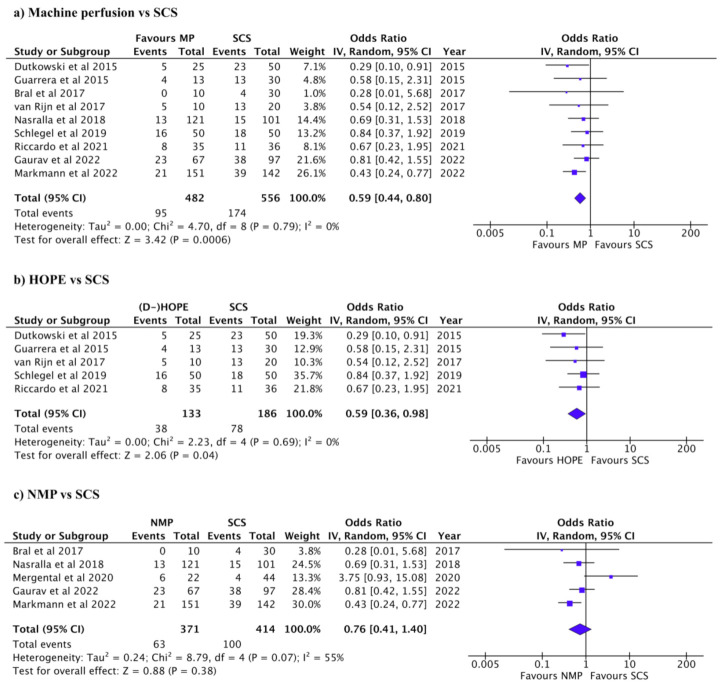
Forest plots for biliary complications. (**a**) Machine perfusion (hypothermic or normothermic) vs. SCS (*P* < 0.001). (**b**) HOPE vs. SCS (*P* = 0.04). (**c**) NMP vs. SCS (*P* = 0.38).

**Figure 3:**
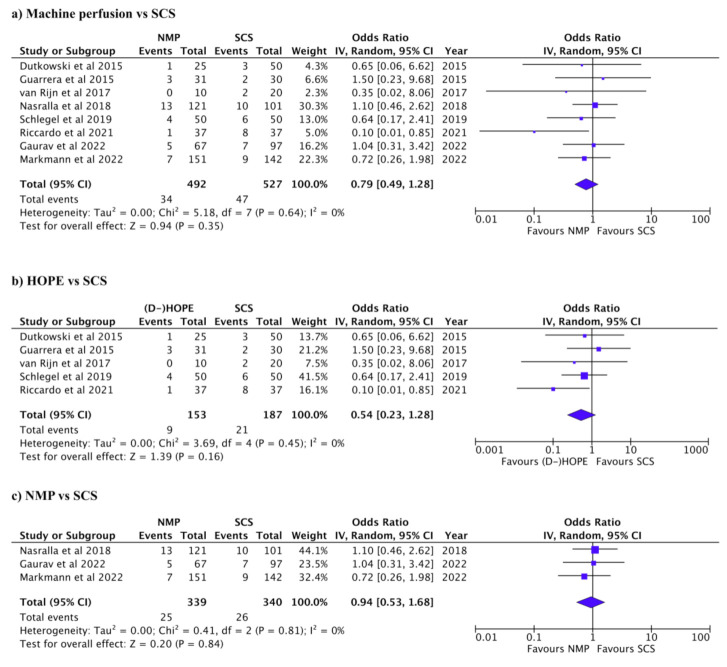
Forest plots for vascular complications. (**a**) Machine perfusion (hypothermic or normothermic) vs. SCS (*P* = 0.35). (**b**) HOPE vs. SCS (*P* = 0.16). (**c**) NMP vs. SCS (*P* = 0.84).

**Figure 4:**
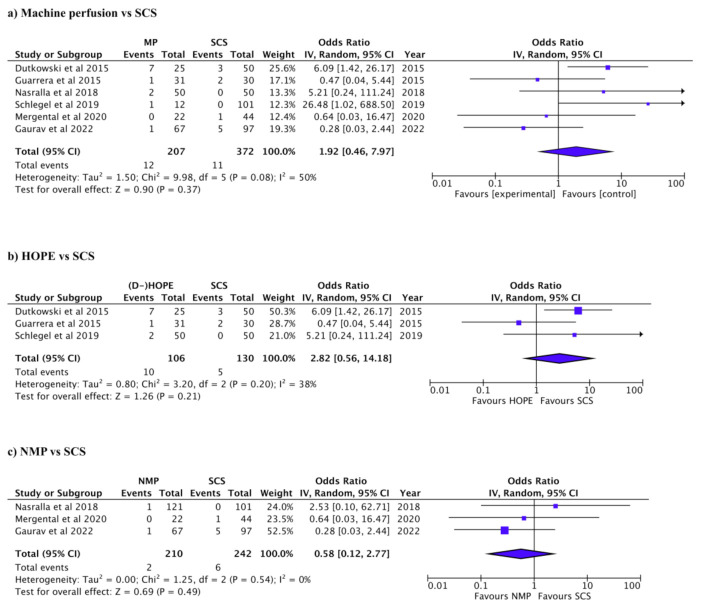
Forest plots for primary non-function. (**a**) Machine perfusion (hypothermic or normothermic) vs. SCS (*P* = 0.37). (**b**) HOPE vs. SCS (*P* = 0.21). (**c**) NMP vs. SCS (*P* = 0.49).

Biliary and vascular complications and primary non-function are summarized in Table 3. Biliary complications were reported in 269/1038 patients in 10 studies. The incidence of biliary complications was higher in SCS than in MP (Pooled OR: 0.59, 95% CI: 0.44–0.80, *p* < 0.001, *I*^2^: 0%, Figure 2a) [7,11,18,20,27,28,29,30,31,32]. When comparing HOPE to SCS, biliary complications were higher in SCS (Pooled OR: 0.59, 95% CI: 0.36–0.98, *p* = 0.04, *I*^2^: 0%, Figure 2b) [18,20,28,29,31]. There was no significant difference in biliary complications between NMP and SCS (Pooled OR: 0.76, 95% CI: 0.41–1.40, *p* = 0.38, *I*^2^: 55%, Figure 2c) [7,11,27,30,32]. Vascular complications were reported in 81/1019 patients in 8 studies [11,18,20,28,29,30,31,32]. There was no significant difference in vascular complications between NM and SCS (Pooled OR: 0.79, 95% CI: 0.49–1.28, *p* = 0.35, *I*^2^: 0%, Figure 3a) [11,18,20,28,29,30,31,32]. There was no significant difference in vascular HOPE and SCS (Pooled OR: 0.54 95% CI: 0.2–1.28, *p* = 0.16, *I*^2^: 0%, Figure 3b) [18,20,28,29,31], nor between NMP and SCS (Pooled OR: 0.94, 95% CI: 0.53–1.68, *p* = 0.84, *I*^2^: 0%, Figure 3c) [7,11,27,30,32]. Vascular complications were not reported in two of the studies [7,27]. PNF was reported in 23/579 patients in six studies [7,11,18,29,30,31]. There was no significant difference in PNF between NM and SCS (Pooled OR: 1.92, 95% CI: 0.46–7.97, *p* = 0.37, *I*^2^: 50%, Figure 4a) [7,11,18,29,30,31]. PNF was also not significantly different between HOPE and SCS, nor between NMP and SCS; (Pooled OR: 2.82, 95% CI: 0.56–14.18, *p* = 0.21, *I*^2^: 38%, Figure 4b) [18,29,31] and (Pooled OR: 0.58, 95% CI: 0.12–2.77, *p* = 0.49, *I*^2^: 0%, Figure 4c) [7,11,30], respectively.

### 3.3. Graft and Patient Survival after Liver Transplant

**Figure 5:**
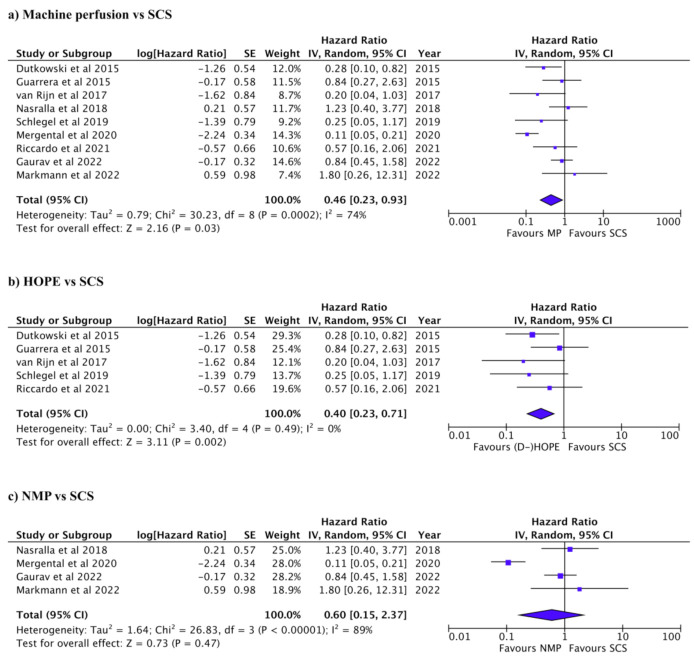
Forest plots for graft survival. (**a**) Machine perfusion (hypothermic or normothermic) vs. SCS (*P* = 0.03). (**b**) HOPE vs. SCS (*P* = 0.02). (**c**) NMP vs. SCS (*P* = 0.47).

**Figure 6:**
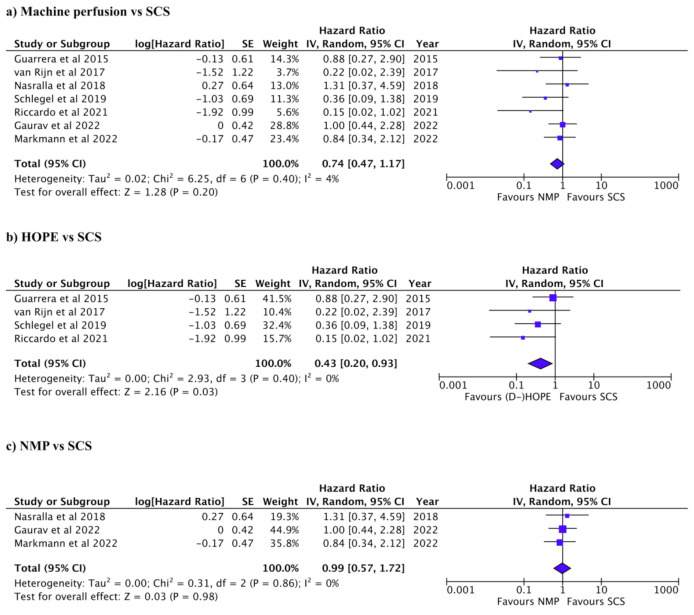
Forest plots for patient survival. (**a**) Machine perfusion (hypothermic or normothermic) vs. SCS (*P* = 0.20). (**b**) HOPE vs. SCS (*P* = 0.03). (**c**) NMP vs. SCS (*P* = 0.98).

The graft and patient survival rates for each of the studies are summarized in Table 4 and Table 5. Re-transplantation was reported in 68/566 patients in five studies (Pooled OR: 0.43, 95% CI: 0.23–0.83, *p* = 0.01, *I*^2^: 0%, Figure S1) [11,20,29,30,31]. Reported 1-year graft survival ranged between 81 and 98% in MP and 69 and 99% in SC. Reported 1-year patient survival ranged between 80 and 100% in the MP and between 80 and 97% in SCS. Graft and patient survival were compared between HOPE and SCS and between NMP and SCS. Graft survival was significantly better in the MP group than SCS (pooled HR: 0.46, 95% CI: 0.23–0.93, *p* = 0.03, *I*^2^: 74%, Figure 5a) [7,11,18,20,28,29,30,31,32]. HOPE was associated with reduced graft loss compared to SCS (pooled HR: 0.40, 95% CI: 0.23–0.71, *p* = 0.002, *I*^2^: 0%, Figure 5b) [18,20,28,29,31]. Graft was slightly favorable in NMP compared to SCS but not statistically significant (pooled HR: 0.60, 95% CI: 0.15–2.37, *p* = 0.47, *I*^2^: 89%, Figure 5c) [7,11,30,32]. There was no significant difference in patient survival between MP and SCS (pooled HR: 0.74, 95% CI: 0.47–1.17, *P* = 0.20, *I*^2^: 4%, Figure 5a) [11,18,20,28,30,31,32]. Patient survival was significantly better in HOPE than SCS (pooled HR: 0.43, 95% CI: 0.20–0.93, *p* = 0.03, *I*^2^: 0%, Figure 6b) [18,20,28,31]. There was no significant difference in patient survival between NMP and SCS (pooled HR: 0.99, 95% CI: 0.57–1.72, *p* = 0.98, *I*^2^: 0%, Figure 6c) [11,30,32]. Funnel plots for studies included in the various analyzes are provided in the supplement; HOPE vs SCS in Figure S2, NMP vs SCS in Figure S3 and MP vs SCS in Figure S4.

## 4. Discussion

Given the increasing demand for donor livers, the gap between supply and demand has kept widening. Several approaches have been taken to try to address this issue. One of which has been the use of ECD organs [33,34]. However, ECD organs are often discarded due to being suboptimal. Secondly, marginal livers are associated with less optimal postoperative outcomes than standard-criteria donor organs. Numerous transplant centers have explored the use of machine perfusion to rescue discarded livers [7,8,35]. The utilization of machine perfusion, however, extends beyond the rescue of discarded organs, and studies have investigated the possibility of replacing SCS with NMP or HOPE/D-HOPE [11,12,18,20,29]. Based on current literature, machine perfusion is associated with more favorable postoperative outcomes. However, there appears to be some difference in the postoperative outcomes of HOPE/D-HOPE vs. SCS and those of NMP vs. SCS.

Both graft and patient survival in liver transplant recipients of grafts that underwent HOPE/D-HOPE instead of SCS were significantly better. The improvement in graft survival may be associated with reduced ischemia-reperfusion injury in grafts that are preserved using HOPE [28,31,36]. The improved patient survival may also be a result of the reduced incidence of postoperative complications and the reduced incidence of graft loss in HOPE compared to SCS. In the HOPE subgroup analysis, graft survival favored the HOPE group in all the studies included. The same was true for patient survival. In both analyses, the studies were homogeneous (*I*^2^: 0%).

However, in the studies that compared NMP to SCS, there was no significant difference in graft and patient survival, although graft survival slightly favored NMP. We do note though that based on *I*^2^-statistic, these studies were heterogenous (*I*^2^: 89%). On further investigation, we found graft survival in Mergental et al. [7] to be the outlier (OR: −2.24, SE: 0.34, in favor of NPM). Without their study included in the subgroup analysis, the studies were homogenous (*I*^2^: 0%). This heterogeneity may be a result of the much smaller sample size in this study compared to the other three studies in the analysis. The NPM and SCS group sample sizes were 22 and 44, respectively, in Mergental et al. [7]; 67 and 97, respectively, in Gaurav et al. [30]; 170 and 164, respectively, in Nasralla et al. [11]; and 142 and 151 in Markmann et al. [32].

Based on these results, HOPE/D-HOPE may provide more favorable graft and patient survival outcomes than NMP. However, we cannot provide concrete backing for this deduction. As such, it should be interpreted as a bird’s-eye-view takeaway from the findings, which merit further investigation.

In a pooled analysis of machine perfusion (NMP and HOPE/D-HOPE) vs. SCS, graft survival was significantly better in the machine perfusion group (*p* = 0.03). However, the studies were significantly heterogeneous (*I*^2^: 74%). The heterogeneity here is most likely a result of the different methods of machine perfusion used in the different studies (HOPE vs. NMP). The patient survival was not significantly better in the machine perfusion group than in SCS, although machine perfusion was slightly favored (*p* = 0.2). Unlike the graft-survival analysis, in this case, the studies were homogenous (*I*^2^: 4%). The patient outcome was mostly affected by the survival results in the studies that used NMP. This is perhaps expected since HOPE and NMP are considered to be distinct graft-preservation techniques. HOPE has been reported to promote mitochondrial functional recovery, increase adenosine triphosphate levels and reduce the donor liver injuring the rewarming phase [16,37]. NPM, on the other hand, has been reported to enable liver metabolism at physiological temperature. NPM has most been used to assess the viability of suboptimal organs [15,38]. Based on current literature, there appears to be no evidence showing a significant benefit of NPM in improving the quality of suboptimal livers. Furthermore, NPM machines have been reported to be technically challenging and prone to human error. Injury to the liver during NPM has a considerably more negative impact on the organ than under HOPE [39].

Since HOPE and NMP may have distinct benefits, with HOPE seemingly being more beneficial to mitigating reperfusion injury, and NPM to allowing for viability testing, some centers are now investigating the combination of HOPE and NPM [40], while some are looking at sub-normothermic machine perfusion [12,14]. We are yet to see whether the sequential use of HOPE followed by NMP can yield much more positive postoperative outcomes coupled with the potential for rescuing marginal and suboptimal organs than may have otherwise been discarded.

We found a similar situation with respect to biliary complications. HOPE had a significantly lower incidence of biliary complications than SCS (*p* = 0.04, *I*^2^: 0%). However, the difference was not significant for NPM vs. SCS, and yet again, the studies were heterogeneous (*I*^2^: 55%). As with graft survival in the studies that compared NPM to SCS, Mergental et al. [7] seems to be the source of the heterogeneity. Analysis without this study included is homogenous with *I*^2^ of 0%. We did, however, find biliary complications to be lower in machine perfusion as a whole vs. SCS (*p* < 0.001, *I*^2^: 0%). For the other postoperative outcomes we analyzed (PNF and vascular complications), there were no significant differences between HOPE and SCS, nor between NMP and SCS.

We could not conduct a detailed analysis of the potential mediating and confounding factors that may have impacted graft and patient survival in the included studies due to patient data availability limitations. However, this is an important aspect of survival analysis. Graft survival in liver transplant patients may be affected by male recipient–female donor sex mismatch, recipient blood group, number of transplantations, advanced donor age, pre-existing portal vein thrombosis and prolonged cold ischemia time [41,42,43]. Patient survival may be influenced by the need for re-transplantation, graft rejection, advanced donor age and prolonged cold ischemia time [42]. To the best of our knowledge, at the time of writing, there is no published study directly comparing HOPE/D-HOPE to NMP. We believe that a standardized multi-center, large sample-size study comparing the two methods and analyzing the potential mediating and confounding factors would be of considerable significance to our understanding of these approaches to donor liver preservation.

The limitations of this study include relatively small sample sizes in some of the studies included. However, since the transplantation of machine-perfused livers is currently being investigated at a limited number of centers, the sample size limitation is still unavoidable. This will undoubtedly change as more liver transplantation centers adopt machine perfusion. Heterogeneity may also have had some impact on the results, especially in the NMP subgroup. In either case, the source of the heterogeneity was a single study whose sample size was much smaller compared to the other studies in the analysis. There may also be limitations due to the inclusion or exclusion bias. There may also be differences in surgical experience at different centers and protocols for HOPE and NMP in the different studies. For all the studies, survival data and hazard ratios were extracted and calculated using the method described by Tierney et al. [26] in their paper. The process of extracting this data may introduce some inaccuracy; however, we think this is mostly negligible since almost all studies tended to favor the research group. The survival rates reported were short-term survival; therefore, for long-term graft and patient survival, further studies are needed.

## 5. Conclusions

Machine perfusion is gaining more interest in donor liver preservation and viability testing for marginal/suboptimal organs. In reported studies, HOPE/D-HOPE has been associated with improved graft and patient survival and reduced biliary complications. NMP has been reported to be helpful in the viability evaluation and rescue of marginal livers. Therefore, HOPE/D-HOPE and NMP are promising alternatives to SCS for donor liver preservation. They may help address the widening gap between the demand for and availability of donor livers by enabling the rescue and transplantation of marginal livers.

## Figures and Tables

**Table 1:** Quality assessment based on the downs and black checklist.

References	Reporting	External Validity	Internal Validity (Risk of Bias)	Internal Validity (Confounding)	Power	Total Points
Dutkowski et al., 2015	10	3	5	3	1	22
Guarrera et al., 2015	9	2	5	2	1	19
Bral et al., 2017	8	3	6	2	1	20
Van Rijn et al., 2017	8	3	6	4	1	22
Nasralla et al., 2018	9	3	6	4	1	23
Schlegel et al., 2019	8	3	5	4	1	21
Mergental et al., 2020	8	3	5	2	1	19
Riccardo et al., 2021	7	3	5	1	1	17
Gaurav et al., 2022	8	3	5	1	1	18
Markmann et al., 2022	8	3	5	3	1	20
Maximum score	11	3	7	6	5	32

**Table 2:** Study characteristics.

References	n		Age		MELD		CIT		Perfusion Time
	HOPE	SCS	HOPE	SCS	HOPE	SCS	HOPE	SCS	
Dutkowski et al., 2015	25	50	60 (57–64)	56 (49–59)	13 (9–15)	16 (10–21)	188 (141–264)	395 (349–447)	317 (280–391)
Guarrera et al., 2015 *	31	30	57.5 ± 8	58.4 ± 9.6	19.5 ± 5.9	21.4 ± 6.3	553 ± 96	516 ± 114	228 ± 54
Van Rijn et al., 2017	10	20	57 (54–62)	52 (42–60)	16 (15–22)	22 (17–27)	-	503 (476–526)	126 (123–135)
Schlegel et al., 2019	50	50	58 (56–62)	57 (51–61)	11 (8–14)	11.8(8.5–15.8)	264 (210–312)	282 (258–318)	120 (96–144)
Riccardo et al., 2021	37	37	58 (37–70)	56 (38–66)	9 (6–25)	13 (6–19)	411 (330–660)	390 (240–583)	120 (42–380)
**References**	**n**		**Age**		**MELD**		**CIT**		**Perfusion Time**
	**NMP**	**SCS**	**NMP**	**SCS**	**NMP**	**SCS**	**NMP**	**SCS**	
Bral et al., 2017	10	30	53(28–67)	59(43–69)	13 (9–32)	19 (7–34)	167 (95–293)	233 (64–890)	690 (198–1350)
Nasralla et al., 2018	121	101	55(48–62)	55(48–62)	13 (10–18)	14 (9–18)	126 (106.5–143)	465 (375–575)	547.5(372.5–710.5)
Mergental et al., 2020	22	44	56(46–65)	-	12 (9–16)	-	452 (389–600)	-	587 (450–705)
Gaurav et al., 2022	67	97	59(51–63)	56(50–62)	14 (10–18)	16 (13–20)	396 (346–441)	430 (397–474)	460 (330–569)
Markmann et al., 2022 *	151	142	57 ± 10.3	58.4 ± 10.1	28.4 ± 6.9	28 ± 5.7	175. 4 ± 43.5	338.8 ± 91.5	276.6 ± 117.4

MELD: Model For End-Stage Liver Disease. CIT: Cold Ischemia Time. HOPE: Hypothermic oxygenated machine perfusion. SCS: Static Cold Storage; NMP: Normothermic Machine Perfusion. * values were reported as mean ± standard deviation. Elsewhere, values were reported as median (range).

**Table 3:** Postoperative complications.

References	Biliary Complications			Vascular Complications			PNF		
	Total	MP	SCS	Total	MP	SCS	Total	MP	SCS
Dutkowski et al., 2015	28	5	23	4	1	3	10	7	3
Guarrera et al., 2015	17	4	13	5	3	2	3	1	2
Bral et al., 2017	4	0	4	-	-	-	-	-	-
Van Rijn et al., 2017	18	5	13	2	0	2	-	-	-
Nasralla et al., 2018	28	13	15	23	13	10	2	2	0
Schlegel et al., 2019	34	16	18	10	4	6	1	1	0
Mergental et al., 2020	-	-	-	-	-	-	1	0	1
Riccardo et al., 2021	19	8	11	9	1	8	-	-	-
Gaurav et al., 2022	61	23	38	12	5	7	6	1	5
Markmann et al., 2022	60	21	39	16	7	9	-	-	-

MP: Machine Perfusion; SCS: Static Cold Storage. PNF: Primary Non-Function.

**Table 4:** Graft survival.

References		Proportion (%) Graft Survival			
		6 months		1 Year	
		MP	SCS	MP	SCS
Dutkowski et al., 2015		-	-	90	69
Guarrera et al., 2015		-	-	81	80
Bral et al., 2017		80	100	-	-
Van Rijn et al., 2017		100	80	100	67
Nasralla et al., 2018		-	-	95	96
Schlegel et al., 2019		-	-	90	82
Mergental et al., 2020		-	-	86.4	86.4
Riccardo et al., 2021		-	-	91.8	83.8
Gaurav et al., 2022		90	87	75	83
Markmann et al., 2022		99	99	98	99

**Table 5:** Patient survival.

References		Proportion (%) Patient Survival	
		1 Year	
		MP	SCS
Dutkowski et al., 2015		-	-
Guarrera et al., 2015		84	80
Bral et al., 2017		100	85
Van Rijn et al., 2017		100	67
Nasralla et al., 2018		95	97
Schlegel et al., 2019		98	86
Mergental et al., 2020		100	95.5
Riccardo et al., 2021		100	91.8
Gaurav et al., 2022		80	94
Markmann et al., 2022		94	93.7

## Data Availability

Not applicable.

## Supplementary Materials

The following are available online at https://www.mdpi.com/article/10.3390/jcm12010235/s1, Figure S1: Forest plots for re-transplantation in Machine Perfusion vs. Static Cold Storage, Figure S2: Funnel plots for HOPE vs. Static Cold Storage, Figure S3: Funnel plots for Normothermic Machine Perfusion vs. Static Cold Storage, Figure S4: Funnel plots for Machine Perfusion vs. Static Cold Storage
