# Supplementary figures and images for: A Meta-Analysis and Systematic Review of Normothermic and Hypothermic Machine Perfusion in Liver Transplantation
Source: J Clin Med. 2022 Dec 28;12(1):235. doi: 10.3390/jcm12010235 (PMC9820958; doi:10.3390/jcm12010235)

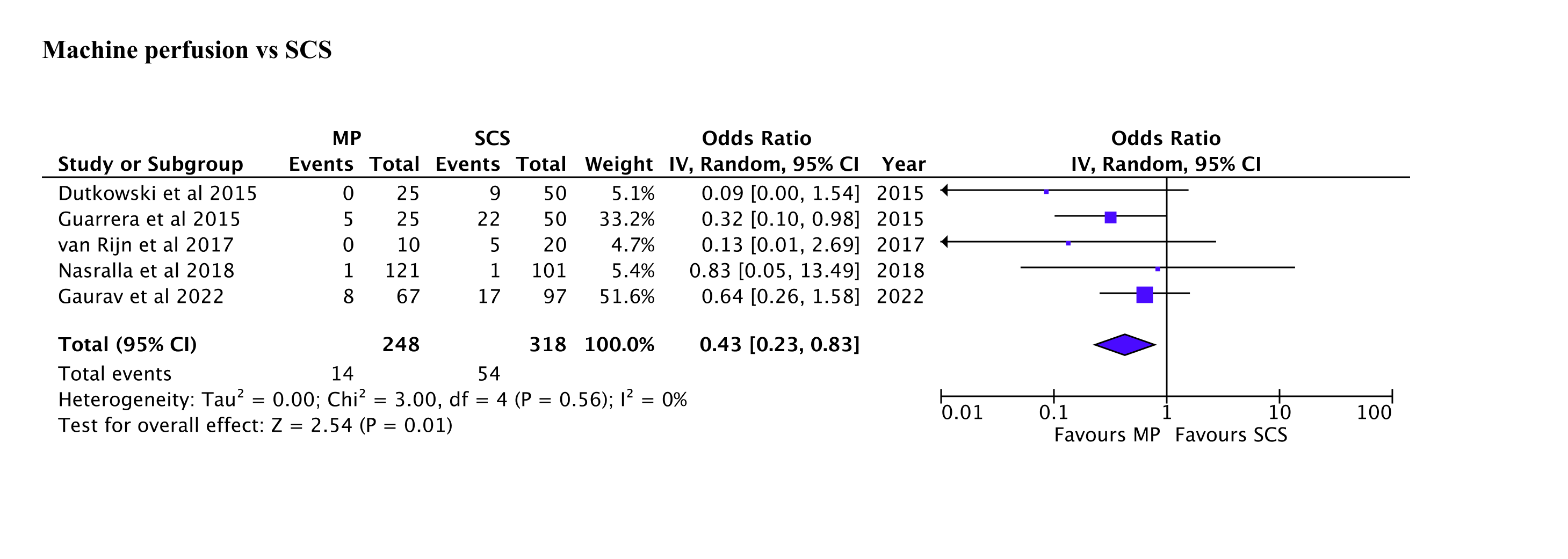

Supplement: Supplementary file 1 [file jcm-12-00235-s001.zip › Figure S1.png]

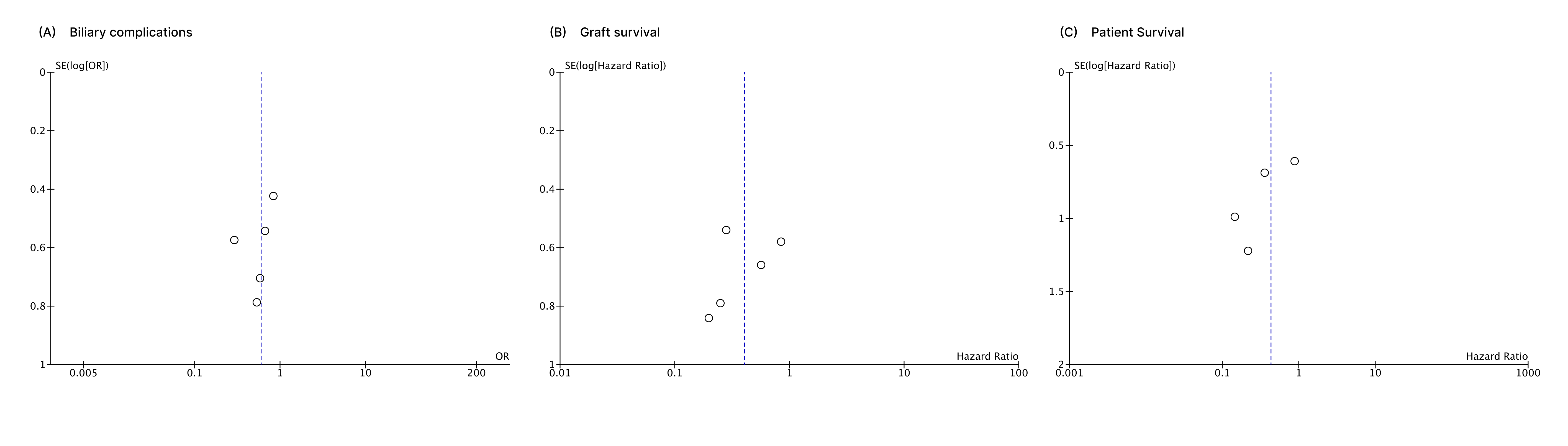

Supplement: Supplementary file 1 [file jcm-12-00235-s001.zip › Figure S2.png]

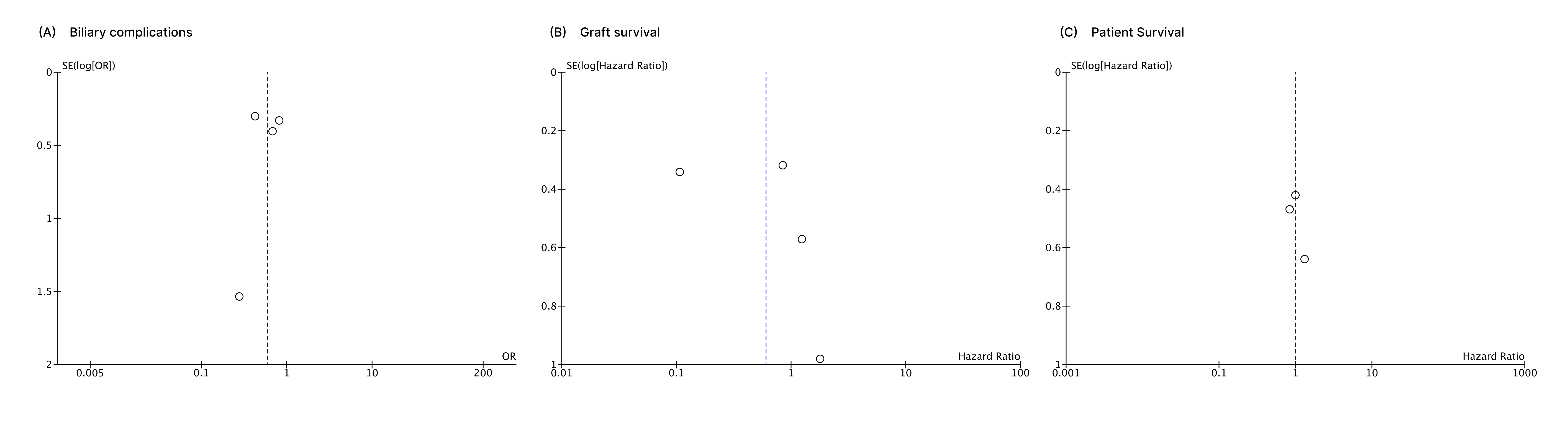

Supplement: Supplementary file 1 [file jcm-12-00235-s001.zip › Figure S3.png]

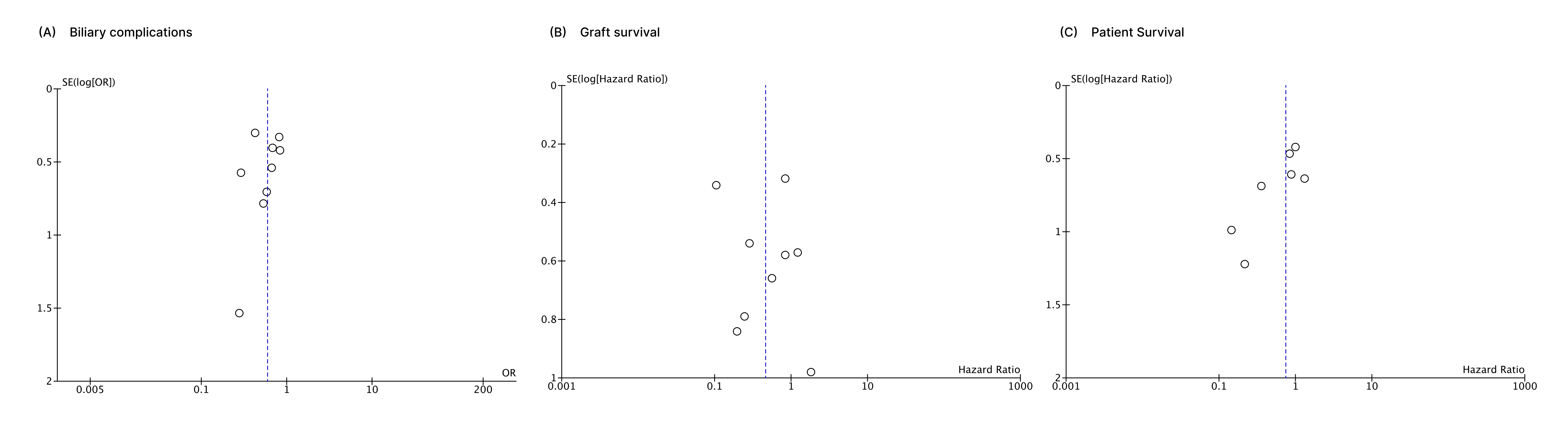

Supplement: Supplementary file 1 [file jcm-12-00235-s001.zip › Figure S4.png]
